# Normative reference values for the 20 m shuttle‐run test in a population‐based sample of school‐aged youth in Bogota, Colombia: the FUPRECOL study

**DOI:** 10.1002/ajhb.22902

**Published:** 2016-08-08

**Authors:** Robinson Ramírez‐Vélez, Adalberto Palacios‐López, Daniel Humberto Prieto‐Benavides, Jorge Enrique Correa‐Bautista, Mikel Izquierdo, Alicia Alonso‐Martínez, Felipe Lobelo

**Affiliations:** ^1^ Centro de Estudios en Medición de la Actividad Física (CEMA), Escuela de Medicina y Ciencias de la Salud, Universidad del RosarioBogotáD.CColombia; ^2^ Grupo GICAEDS, Facultad de Cultura Física, Deporte y Recreación, Universidad Santo TomásBogotáD.CColombia; ^3^ Department of Health SciencesPublic University of NavarraNavarraSpain; ^4^ Hubert Department of Global HealthRollins School of Public Health, Emory UniversityAtlantaGeorgia

**Keywords:** cardiorespiratory fitness, children, adolescent, percentiles, normative data

## Abstract

**Objectives:**

Our aim was to determine the normative reference values of cardiorespiratory fitness (CRF) and to establish the proportion of subjects with low CRF suggestive of future cardio‐metabolic risk.

**Methods:**

A total of 7244 children and adolescents attending public schools in Bogota, Colombia (55.7% girls; age range of 9–17.9 years) participated in this study. We expressed CRF performance as the nearest stage (minute) completed and the estimated peak oxygen consumption (V˙O_2peak_). Smoothed percentile curves were calculated. In addition, we present the prevalence of low CRF after applying a correction factor to account for the impact of Bogota's altitude (2625 m over sea level) on CRF assessment, and we calculated the number of participants who fell below health‐related FITNESSGRAM cut‐points for low CRF.

**Results:**

Shuttles and V˙O_2peak_ were higher in boys than in girls in all age groups. In boys, there were higher levels of performance with increasing age, with most gains between the ages of 13 and 17. The proportion of subjects with a low CRF, suggestive of future cardio‐metabolic risk (health risk FITNESSGRAM category) was 31.5% (28.2% for boys and 34.1% for girls; X^2^ *P* = .001). After applying a 1.11 altitude correction factor, the overall prevalence of low CRF was 11.5% (9.6% for boys and 13.1% for girls; X^2^ *P* = .001).

**Conclusions:**

Our results provide sex‐ and age‐specific normative reference standards for the 20 m shuttle‐run test and estimated V˙O_2peak_ values in a large, population‐based sample of schoolchildren from a large Latin‐American city at high altitude.

## 1 Introduction

Cardiorespiratory fitness (CRF) is a direct indicator of an individual's physiological status and reflects the overall capacity of the cardiovascular and respiratory systems (Castro‐Piñero et al., 2012). There are a number of cross‐sectional studies showing that low CRF in youth is associated with a higher cardio‐metabolic risk including higher fasting blood glucose, abdominal obesity, triglycerides, and blood pressure, independent of socio‐demographic factors, diet, and physical activity (Andersen et al., 2006; Lobelo, Pate, Dowda, Liese, & Daniels, 2010; Ruiz et al., 2006). In addition, longitudinal studies have shown that a healthy CRF in childhood and adolescence is associated with a healthier cardio‐metabolic profile later in life (Ortega, Ruiz, Castillo, & Sjostrom, 2008). These findings have been replicated in clinical adult populations with diabetes mellitus, hypertension, metabolic syndrome, and several types of cancer (LaMonte & Blair, 2006). In light of this evidence, it is of great concern that in recent decades, CRF appears to have declined in children and adolescents worldwide (Catley & Tomkinson, 2013; Garber, Sajuria, & Lobelo, 2014). These trends have been observed against a background of increased, decreased, or stable body mass index (BMI) within the same populations (Olds, Tomkinson, Leger, & Cazorla, 2006).

Sex‐ and age‐specific normative data for CRF in youth have been published (Barnett, Bacon‐Shone, Tam, Leung, & Armstrong, 1995; Eisenmann & Malina, 2002; Ortega et al., 2011; Pate, Wang, Dowda, Farrell, & O'neill, 2006; Twisk, Kemper, & van Mechelen, 2002). However, the majority of the published aerobic fitness reference values are for schoolchildren from high income countries in North America (Carrel et al., 2012; Pate et al., 2006; Tremblay et al., 2010), Asia/Oceania (Gürsel, Sonel, Gök, & Yalçin, 2004; Tomkinson, Léger, Olds, & Cazorla, 2003; Tomkinson, Olds, Kang, & Kim, 2007), and Europe (Gulías‐González, Sánchez‐López, Olivas‐Bravo, Solera‐Martínez, & Martínez‐Vizcaíno, 2014; Haugen, Høigaard, & Seiler, 2014; Roriz De Oliveira, Seabra, Freitas, Eisenmann, & Maia, 2014; Sandercock, Voss, Cohen, Taylor, & Stasinopoulos, 2012). There is a scarcity of reference values for children using harmonized measures of physical fitness in Latin America (Aguilar et al., 2011; González et al., 2014) and other low‐middle income countries undergoing rapid epidemiologic and nutrition transitions (Wachira, Muthuri, Tremblay, & Onywera, 2014), making it impossible to evaluate secular trends within these regions and identify high risk groups for which risk reduction interventions should be prioritized. In particular, no population‐based studies have been conducted to assess CRF for youth living at high altitude (over 2000 m over sea level). This is important because it is estimated that more than 16.5 million youth between the ages of 10‐ and 20‐year old live at such altitudes in the Andes in Latin America and in mountain ranges in Central America, Europe, Asia and Africa, for which CRF normative values may need to be created or adjusted.

Therefore, the objectives of this study were threefold. First is to present normative reference values for CRF in a population‐based sample of 9‐ to 17‐year old school‐aged youth in Bogota, Colombia as estimated via the 20 m Shuttle‐run test. Second is to establish the proportion of subjects whose aerobic capacity is indicative of future cardiovascular risk based on previously validated health‐related standards that define low CRF. Finally, to investigate the prevalence of low CRF after applying a correction factor previously suggested in the literature, to account for the impact of Bogota's altitude (2625 m over sea level) on CRF assessment.

## 2 Methods

### 2.1 Study population

In Colombia, measures of weight and physical activity have been added to youth health monitoring systems by the government (ICBF, 2010) and research institutions. Recently (2015), physical fitness assessment was added to the FUPRECOL study (*in Spanish* ASOCIACIÓN DE LA **FU**ERZA **PRE**NSIL CON MANIFESTACIONES DE RIESGO CARDIOVASCULAR TEMPRANAS EN NIÑOS Y ADOLESCENTES **COL**OMBIANOS). The FUPRECOL study seeks to establish the general prevalence of cardiovascular risk factors (anthropometric, metabolic, and genetic markers) in the study population (children and adolescents aged 9 to 17.9 years living in Bogota, Colombia) (Prieto‐Benavides, Correa‐Bautista, & Ramírez‐Vélez, 2015; Ramírez‐Vélez, Rodrigues‐Bezerra, Correa‐Bautista, Izquierdo, & Lobelo, 2015; Rodríguez‐Bautista, Correa‐Bautista, González‐Jiménez, Schmidt‐RioValle, & Ramírez‐Vélez, 2015) and examine the relationships between physical fitness levels and cardio‐metabolic risk factors. The FUPRECOL study assessments were conducted during the 2014–2015 school year. A total of 8000 students were considered for physical fitness evaluation from all regions and 20 municipalities (localidades) in the capital district of Bogota. The sample consisted of children (ages 9 to 11.9 years; *n* = 3373) and adolescents (ages 12 to 17.9 years; *n* = 3871). Erroneous data entry (*n* = 86), student disability (*n* = 50), student temporary illness or injury (*n* = 108), student chronic illness (*n* = 298), or absenteeism (*n* = 214), limited the analytical sample to 7244 students. All schoolchildren were of low‐middle socioeconomic status (SES, 1–3 in a scale 1–6 defined by the Colombian government) and enrolled in public elementary and high schools (grades 5 through 11) in the capital district of Bogota, Cundinamarca Department in the Andean region. This region is located at approximately 4°35′56″N 74°04′51″W and at an elevation of approximately 2625 m (min: 2500; max: 3250) above sea level. Bogota is considered an urban area, with approximately 7,862,277 inhabitants (DANE, 2007). A convenience sample of volunteers was recruited and grouped by sex and age based on 1‐year intervals (9 groups total). Power calculations were based on the mean CRF values from the first 200 participants recruited (range: 35–45 ml kg^−1^ min^−1^), with a group standard deviations (SD) of approximately 5.2 ml kg^−1^ min^−1^. The significance level was set to 0.05, and the required power was set to at least 0.80. The sample size was estimated to be approximately 200 to 400 participants per sex‐ and age. Exclusion factors included clinical diagnosis of cardiovascular disease, diabetes mellitus 1 and 2, pregnancy, use of alcohol or drugs, not having lived in Bogota for at least 1 school year. Exclusion from the study was made effective *a posteriori*, without the students being aware of their exclusion to avoid any undesired situations.

### 2.2 Data collection

Anthropometric variables were measured by a Level 2 anthropometrist certified by the International Society for the Advancement of Kinanthropometry (ISAK) in accordance with the ISAK guidelines (Marfell‐Jones, Olds, & Stewart, 2006). Variables were collected at the same time in the morning, between 7:00 and 10:00 a.m., following an overnight fast. Body mass of the subjects was measured when the subjects were in underwear and did not have shoes on, using electronic scales (Tanita® BC544, Tokyo, Japan) with a low technical error of measurement (TEM = 0.51%). Height was measured using a mechanical stadiometer platform (Seca^®^ 274, Hamburg, Germany; TEM = 0.01%). BMI was calculated as the body weight in kilograms divided by the square of the height in meters. Weight status categories were defined as having a BMI above the age and sex‐specific thresholds of the IOTF (Cole, Bellizzi, Flegal, & Dietz, 2000). Waist circumference was measured at the midpoint level of the mid‐axillary line between the 12th rib head and the superior anterior iliac spine using a tape measure (Ohaus^®^ 8004‐MA, New Jersey). Sexual maturation was classified based on Tanner staging (Tanner & Whitehouse, 1976), which uses self‐reported puberty status to classify participants into stages I to V (Matsudo & Matsudo, 1994). Each volunteer entered an isolated room where they categorized the development of their own genitalia (for boys), breasts (for girls), armpits (for boys) and pubic hair (for both genders) using a set of images exemplifying the various stages of sexual maturation. The reproducibility of our data reached *R* = 0.78. The data were recorded on paper by the FUPRECOL evaluators.

### 2.3 Cardiorespiratory fitness

Testing procedures were consistent with international guidelines for school‐based fitness assessment (Universidad de Granada, 2007). At each school, a team of trained FUPRECOL evaluators administered the tests in partnership with the schooĺs physical education instructor. Testing was conducted in the school gymnasium or in areas where a hard surface was available. The 20mSRT was administered as described by Leger et al (Leger, Mercier, Gadoury, & Lambert, 1988). Participants jogged or ran in a straight line between two lines 20 m apart while keeping pace with pre‐recorded audio signals. The initial speed was 8.5 km/hour and increased by 0.5 km/hour each minute. The test was terminated if the participant failed to reach the end lines in time with the audio signals on two consecutive occasions or when the subject stopped because of self‐reported fatigue. Results were recorded to the nearest stage (minute) completed. The equation of Leger et al. (1988) was used to estimate the V˙O_2peak_. To calculate the V˙O_2peak_ from the result of the 20 m shuttle‐run test score, age (A; in years), and the final speed (S; running speed at the last completed level (km·h^−1^) = 8 + 0.5 per stage number, in km·h^−1^) were entered into the following formula (*r* = 0.7; for children and adolescents, from 8–19 years) V˙O_2peak_ (ml kg^−1^ min^−1^) = 31.025 + 3.238 x S ‐ 3.248 x A + 0.1536 × S × A. The reliability and validity of this test has been widely documented (Leger, Lambert, Goulet, Rowan, & Dinelle, 1984; Liu, Plowman, & Looney, 1992) and is considered a test of choice for population‐based CRF assessments for schoolchildren (Ramírez‐Vélez et al., 2015). All tests were conducted by a trained research team that provided standardized encouragement for participants during all test phases. CRF measurements in a subsample (*n* = 229, median age = 12.8 ± 2.4 y, 46.2 ± 12.4 kg, 1.50 ± 0.1 m, 19.9 ± 3.1 kg/m^2^) were recorded to ensure reproducibility on the day of the study. The reproducibility of our data was *R* = 0.84. Intra‐rater reliability was assessed by determining the intraclass correlation coefficient (ICC = 0.96, CI 95% 0.95 to 0.97).

### 2.4 CRF and future cardio‐metabolic risk

In the 1990s, FITNESSGRAM^®^ established sex‐ and age‐specific CRF cutoff values for adolescents that were known as the Healthy Fitness Zones (Cureton & Warren, 1990). The Healthy Fitness Zones were designed to represent the lowest levels of CRF that were linked to adequate functional and/or health‐related outcomes in adolescents (Garber et al., 2014; Sandercock et al., 2012). We calculated the number of participants in each age‐sex group who might be considered to have low CRF levels according to recently‐updated FITNESSGRAM^®^ health‐related standards (Welk, Laurson, Eisenmann, & Cureton, 2011). Low CRF was defined using either the cut‐off by sex and age (shuttle‐runs or estimated V˙O_2peak_) listed in the healthy fitness zone (needs improvement and health risk). The FITNESSGRAM^®^ (Welk et al., 2011) has been shown to have cardio‐metabolic health predictive value (Welk et al., 2011), and V˙O_2peak_ cut‐off points were validated against the presence of the metabolic syndrome using nationally representative U.S. data (Lobelo, Pate, Dowda, Liese, & Ruiz, 2009).

All measures were performed twice, except for the 20 m shuttle‐run tests, which were performed only once for the study sample. All standard operating procedures and protocols for CRF testing are available in the manual and videos on the ALPHA project Web site (Universidad de Granada, 2007). Direct and simple language was used for the communication process and the explanation of tests. Additionally, the evaluators provided visual models and examples before performing the test when necessary. Participants did not receive previous training on these tests.

#### 2.4.1 Altitude correction factor

Previous studies have reported VO_2max_ values to be reduced by about 10% for each 1000 m above 1500 m, point where arterial oxygen desaturation is evident despite adequate erythropoietic adaptations (Fulco, Rock, & Cymerman, 1998; Squires & Buskirk, 1982) in altitude‐adjusted individuals. However, other studies have shown a non‐significant (3.6%) decrease in VO_2max_ for untrained adults versus a significant (6.8%) decrease in trained adults at 580 m, indicating that at lower altitudes, untrained adults may not be ventilation‐limited (Gore et al., 1996). In our analysis, we present both raw V˙O_2peak_ values as well as values adjusted by a correction factor of 1.11 to account for the impact of Bogota's altitude (2625 m over sea level) on CRF assessment. We believe that the correction factor we applied is warranted based on the available, although limited, evidence and altitude‐adjusted CRF normative values should be presented.

#### 2.4.2 Ethics statement

The Review Committee for Research on Human Subjects at the University of Rosario [Code Nº CEI‐ABN026‐000262] approved all of the study procedures. A comprehensive verbal description of the nature and purpose of the study and its experimental risks was given to the participants and their parents/guardians. This information was also sent to parents/guardians by mail. Written informed consent was obtained from parents and subjects before participation in the study. The protocol was in accordance with the latest revision of the Declaration of Helsinki and current Colombian laws governing clinical research on human subjects (Resolution 008430/1993 Ministry of health).

### 2.5 Statistical analysis

Anthropometric and CRF characteristics of the study sample are presented as means and SD. Normality of selected variables was verified using histograms and Q‐Q plots. Differences were analyzed by two‐way analysis of variance (ANOVA) or Chi‐square test (X^2^) to explore sex and age differences. Smoothed and specific curves for each age were obtained via a penalized maximum likelihood with the following abbreviations: (1) L (Box‐Cox transformation), (2) M (median), and (3) S (coefficient of variation) (Cole & Green, 1992). The LMS method assumes that the outcome variable has a normal distribution after a Box‐Cox power transformation is applied using the LMS method implemented in the LMSChartMaker Pro Version 2.54, (Medical Research Council, London, UK, http://www.healthforallchildren.com/shop-base/software/lmschartmaker-light/). The appropriate number of degrees of freedom was selected on the basis of deviance, Q‐tests and worm plots, following the suggestions of Royston & Wright (Royston & Wright, 2000). The 3rd, 10th, 25th, 50th, 75th, 90th, and 97th smoothing percentiles were chosen by sex and age for reference values. We used SPSS V. 21.0 software for Windows (SPSS, Chicago, Illinois) for all but the LMS method calculations. Statistical significance was set at *P* < .05

## 3 Results

### 3.1 Descriptive characteristics

Descriptive statistics by sex are shown in Table 1. All anthropometric variables, except BMI, were higher in boys than in girls (*P* < .001). The prevalence of overweight and obesity differed by sex (*P* < .001). Two‐way ANOVA tests showed that V˙O_2peak_ (ml kg^−1^ min^−1^) and shuttles (count) were higher in boys than in girls (*P* < .001). The proportion of subjects with a low CRF suggestive of future cardio‐metabolic risk (health risk FITNESSGRAM standard) was 31.5% (28.2% for boys and 34.1% for girls; X^2^ *P* = .001). After applying an 11% altitude correction factor, the overall prevalence of low CRF was 11.5% (9.6% for boys and 13.1% for girls; X^2^ *P* = .001).

**Table 1:** Characteristics of among a population‐based sample of schoolchildren in Bogota, Colombia [mean (SD) or frequencies (%)]

Characteristics	Boys (*n* = 3211)	Girls (*n* = 4033)	Total (*n* = 7244)
Age (years)	12.9 (2.3)	12.8 (2.4)*	12.8 (2.3)
Body mass (kg)	45.0 (13.0)	44.8 (11.4)**	44.6 (12.3)
Height (m)	1.50 (0.13)	1.47 (0.10)*	1.49 (0.12)
BMI (kg/m^2^)	19.3 (3.3)	20.3 (3.5)*	19.7 (3.4)
Weight status n,(%)			
Underweight	930 (29)	1209 (31)*	2139 (29)
Normal weight	1697 (53)	1828(45)*	3525 (49)
Overweight	405 (13)	658 (16)*	1063 (15)
Obese	179 (6)	338 (8)*	517 (7)
Waist circumference (cm)	66.3 (8.2)	65.0 (8.1)*	65.6 (8.1)
Sexual maturation status n,(%)			
I	244 (8)	710 (18)*	954 (13)
II	988 (31)	960 (24)*	1948 (27)
III	922 (29)	981 (24)*	1903 (26)
IV	833 (26)	1189 (29)*	2022 (28)
V	224 (7)	193 (5)*	417 (6)
V˙O_2peak_ (ml kg^−1^ min^−1^)^a^	42.4 (5.2)*	38.5 (4.8)*	40.2 (5.3)
V˙O_2peak_ altitude‐adjusted (ml kg^−1^ min^−1^)^b^	47.1 (5.8)*	42.7 (5.3)*	44.6 (5.9)
Shuttles (total count)	36.6 (20.0)*	22.3 (11.3)*	28.7 (17.3)
Stage (last completed)	4.4 (2.2)*	2.8 (1.3)*	3.5 (2.0)
Running speed at last completed shuttle (km h^−1^)	9.9 (2.1) *	9.0 (1.8)*	3.5 (2.0)
FITNESSGRAM (fitness zones) n,(%)^c^			
Needs improvement	538 (16.8)*	1039 (25.8)*	1577 (21.8)
Health risk	904 (28.2)*	1373 (34.1)*	2277 (31.5)
FITNESSGRAM (fitness zones) n,(%)^b^			
Needs improvement	268 (8.3)*	573 (14.2)*	841 (11.6)
Health risk	308 (9.6)*	530 (13.1)*	838 (11.5)

*Note*: Frequencies in brackets represent the proportion of the total sample with data for each variable.

Significant between‐sex differences (ANOVA one way test or Chi‐square; *P < .001; **P < .01).

a VO_2peak_ (ml kg^−1^ min^−1^) predicted using the Leger et al. equation (1988).

b V˙O_2peak_ (ml kg^−1^ min^−1^) predicted using the Leger et al. equation (1988) and adjusted by an altitude correction factor (1.11).

c To classify V˙O_2peak_, we used the 2011 FITNESSGRAM^®^ standards and Healthy Fitness Zones (Welk et al., 2011).

### 3.2 Twenty meter shuttle‐run performance and FITNESSGRAM health‐related standards

The age‐sex distribution of 20 m shuttle‐run performance for the study sample is presented in Table 2. Performance is expressed as number of shuttles completed, running speed for the final shuttle completed and predicted V˙O_2peak_ (ml kg^−1^ min^−1^). The prevalence of low CRF (health risk FITNESSGRAM category) was 0% in 9‐ to 11.9‐year‐olds but ranged from 15.4% to 79.2% in other age groups, being particularly high among 15‐ to 17‐year‐old girls and 17‐year‐old boys. The altitude‐adjusted prevalence of low CRF ranged from 0% to 60%, following a similar pattern by sex and age groups.

**Table 2:** Descriptive statistics for 20 m‐shuttle run performance and FITNESSGRAM Health‐related standards among a population‐based sample of schoolchildren in Bogota, Colombia

						**FITNESSGRAM (fitness zones)** c			**FITNESSGRAM Altitude‐Adjusted (fitness zones)** b	
**Sex**	***n***	**Shuttles (total count)**	**Running speed at last completed shuttle (km h^−1^)**	**V˙O_2peak_ (ml kg^−1^ min^−1^)** a	**V˙O_2peak_ Altitude‐adjusted (ml kg^−1^ min^−1^)** b	**Needs improvement**		**Health risk**	**Needs improvement**	**Health risk**
**Boys**										
9 to 9.9	215	20.0 (11.8)	8.6 (2.4)	44.6 (3.2)	49.5 (3.5)	0.0%	0.0%		0.0%	0.0%
10 to 10.9	399	24.2 (13.3)	9.2 (1.2)	43.6 (3.7)	48.4 (4.1)	22.6%	0.0%		0.0%	0.0%
11 to 11.9	408	26.1 (13.5)	9.3 (1.4)	42.4 (4.0)	47.1 (4.4)	23.5%	18.1%		0.0%	0.0%
12 to 12.9	381	29.7 (15.2)	9.6 (1.6)	41.8 (4.6)	46.4 (5.1)	21.0%	26.8%		13.1%	0.0%
13 to 13.9	391	35.8 (16.9)	10.0 (1.9)	42.0 (5.0)	46.6 (5.6)	11.5%	23.0%		13.3%	6.9%
14 to 14.9	434	42.2 (18.9)	10.2 (2.0)	42.4 (5.9)	47.0 (6.5)	17.7%	41.0%		10.4%	14.4%
15 to 15.9	403	47.3 (19.1)	10.4 (2.2)	42.2 (6.2)	46.8 (6.8)	48.1%	19.6%		11.9%	15.4%
16 to 16.9	340	51.4 (19.7)	10.6 (2.4)	41.9 (6.3)	46.5 (6.9)	15.6%	39.4%		11.8%	27.4%
17 to 17.9	240	54.0 (19.9)	10.7 (2.4)	40.9 (6.4)	45.4 (7.1)	14.7%	55.5%		13.9%	20.2%
Total	3211	36.6 (20.1)	9.9 (2.1)	42.4 (5.2)	47.1 (5.8)	16,8%	28,2%		8,3%	9,6%
**Girls**										
9 to 9.9	293	15.8 (8.6)	8.5 (1.9)	43.3 (2.4)	48.0 (2.6)	0.0%	0.0%		0.0%	0.0%
10 to 10.9	628	18.5 (10.0)	8.9 (1.5)	42.0 (2.9)	46.6 (3.2)	36.3%	0.0%		0.0%	0.0%
11 to 11.9	585	19.9 (9.5)	9.1 (1.0)	40.5 (2.9)	45.0 (3.2)	39.1%	25.8%		0.0%	0.0%
12 to 12.9	464	21.4 (9.5)	9.1 (1.5)	39.4 (3.1)	43.7 (3.4)	29.1%	20.7%		20.7%	0.0%
13 to 13.9	429	23.7 (11.6)	9.2 (1.9)	38.5 (4.0)	42.8 (4.4)	28.4%	43.1%		15.4%	0.0%
14 to 14.9	555	25.2 (11.2)	9.3 (1.6)	37.2 (4.0)	41.3 (4.4)	29.0%	36.4%		25.4%	11.0%
15 to 15.9	434	25.0 (11.9)	9.0 (2.3)	35.2 (4.2)	39.1 (4.7)	26.7%	68.2%		16.4%	41.5%
16 to 16.9	383	26.5 (12.0)	9.2 (2.2)	34.1 (4.5)	37.9 (5.0)	18.5%	61.9%		27.2%	34.7%
17 to 17.9	262	28.1 (13.1)	9.0 (2.6)	32.7 (4.8)	36.3 (5.3)	8.5%	79.2%		19.2%	60.0%
Total	4033	22.4 (11.3)	9.0 (1.8)	38.5 (4.8)	42.7 (5.3)	25.8%	34,1%		14,2%	13,1%

*Note*: Data are mean (SD) for continuous variables or (%).

a V˙O_2peak_ (ml kg^−1^ min^−1^) predicted using the Leger et al. equation (1988).

b V˙O_2peak_ (ml kg^−1^ min^−1^) predicted using the Leger et al. equation (1988) and adjusted by an altitude correction factor (1.11).

c To classify V˙O_2peak_, we used the 2011 FITNESSGRAM^®^ standards and Healthy Fitness Zones (Welk et al., 2011).

### 3.3 Normative 20 m shuttle‐run values

**Figure 1:**
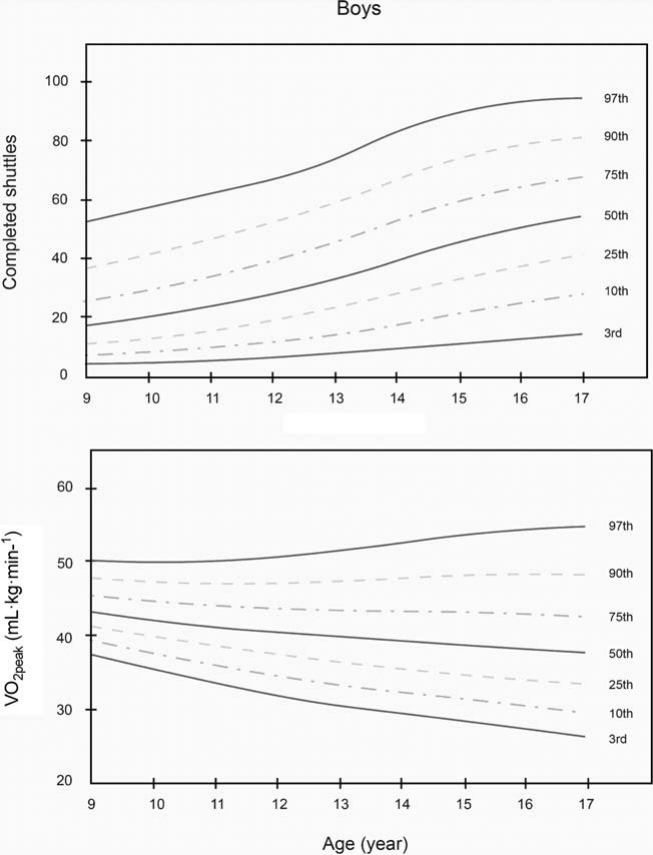
Centile curves for 20 m shuttle run test (Total shuttles completed) and V ˙O_2peak_ (ml kg^−1^ min^−1^) among a population‐based sample of schoolboys in Bogota, Colombia

**Figure 2:**
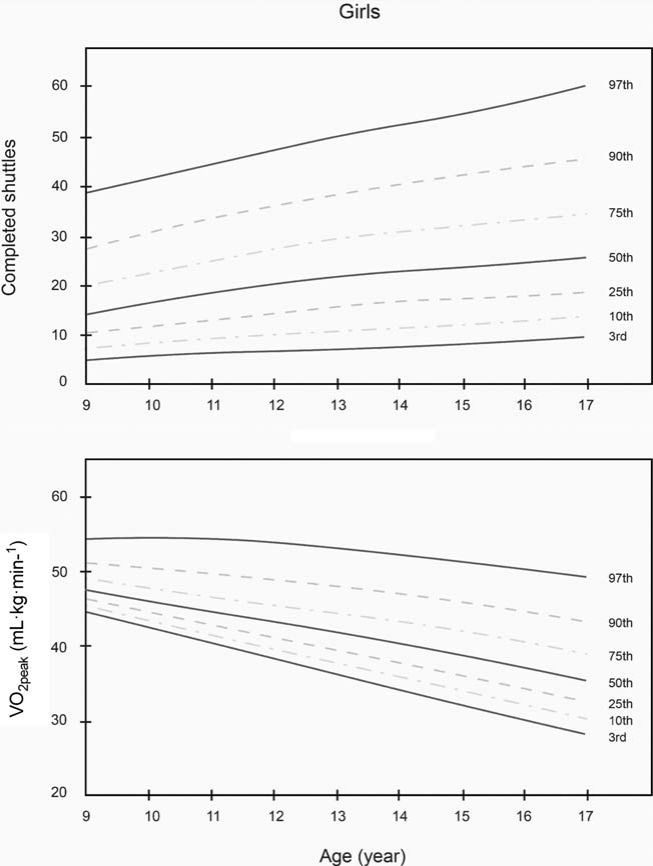
Centile curves for 20 m shuttle run test (Total shuttles completed) and V ˙O_2peak_ (ml kg^−1^ min^−1^) among a population‐based sample of schoolgirls in Bogota, Colombia

Smoothed LMS curves (3rd, 10th, 25th, 50th, 75th, 90th, and 97th percentile) for boys' and girls' performance (shuttles completed and V˙O_2peak_ in ml kg^−1^ min^−1^) are shown in Figures 1 and 2. The equivalent numerical values are available in Tables 3 and 4. Together, these data show that boys performed better on the test at all ages compared with girls. In boys, the 50th percentile of shuttles completed, V˙O_2peak_ and altitude‐adjusted V˙O_2peak_ ranged from 17.5 to 52.0 shuttles, 40.3 to 43.9 ml kg^−1^ min^−1^, and 44.7 to 48.7 ml kg^−1^ min^−1^, respectively. In girls, the 50th percentile ranged from 15.0 to 25.0 shuttles, 31.5 to 43.4 ml kg^−1^ min^−1^, and 35.0 to 48.2 ml kg^−1^ min^−1^, respectively. In boys, there were higher levels of performance across all age groups, with most apparent gains between the ages of 14 and 17. In girls, performance was higher between the ages of 12 and 14, but this difference was more modest. From the ages of 15 to 15.9, test performance was slightly lower in girls (Table 4).

**Table 3:** Smoothed age‐ and sex‐specific percentile values for the 20 m Shuttle run test (Total Shuttles Completed) among a population‐based sample of schoolchildren in Bogota, Colombia

	***n***	**P_3_**	**P_10_**	**P_25_**	**P_50_**	**P_75_**	**P_90_**	**P_97_**
**Boys**								
9 to 9.9	215	5.0	8.0	11.0	16.0	27.0	36.0	43.5
10 to 10.9	399	7.0	9.0	14.0	22.0	32.0	42.0	54.0
11 to 11.9	408	7.0	10.0	15.0	23.5	34.0	47.0	55.5
12 to 12.9	381	8.0	12.0	18.0	27.0	39.0	50.0	63.5
13 to 13.9	391	8.9	14.0	24.0	34.0	46.0	59.0	76.0
14 to 14.9	434	10.1	18.5	28.0	40.0	56.0	66.0	79.9
15 to 15.9	403	12.0	22.0	34.0	48.0	60.0	71.0	81.9
16 to 16.9	340	16.0	25.1	35.3	52.0	66.0	78.0	87.0
17 to 17.9	240	14.0	27.0	40.0	54.0	68.0	80.2	91.3
Total	3211	8.0	12.0	20.0	34.0	50.0	65.0	78.6
**Girls**								
9 to 9.9	293	6.0	8.0	10.0	14.0	19.0	27.0	37.2
10 to 10.9	628	7.0	9.0	12.0	16.0	22.0	32.0	43.0
11 to 11.9	585	8.0	10.0	13.0	18.0	24.0	33.0	44.0
12 to 12.9	464	7.0	10.5	14.0	20.0	27.0	35.0	42.0
13 to 13.9	429	7.9	11.0	15.0	21.0	30.0	41.0	51.0
14 to 14.9	555	10.0	12.0	16.0	23.0	32.0	41.0	51.0
15 to 15.9	434	10.0	12.0	16.0	22.0	30.0	40.0	52.0
16 to 16.9	383	10.0	13.4	17.0	25.0	34.0	43.0	52.0
17 to 17.9	262	12.0	14.0	18.0	25.0	35.0	47.0	60.0
Total	4033	8.0	10.0	14.0	20.0	28.0	37.0	49.0

P = percentile.

**Table 4:** Smoothed age‐ and sex‐specific percentile values for V˙O_2peak_ (ml kg^−1^ min^−1^) among a population‐based sample of schoolchildren in Bogota, Colombia

	***n***	**P_3_**	**P_10_**	**P_25_**	**P_50_**	**P_75_**	**P_90_**	**P_97_**
**Boys**								
9 to 9.9	215	41.1	41.2	41.2	43.5	45.8	48.2	51.6
10 to 10.9	399	39.3	39.3	41.5	43.9	46.4	48.8	51.2
11 to 11.9	408	37.3	37.3	39.8	42.3	44.7	47.4	49.7
12 to 12.9	381	35.4	35.4	37.9	40.5	45.4	48.1	50.6
13 to 13.9	391	33.4	35.9	38.7	41.3	43.9	49.2	51.8
14 to 14.9	434	31.5	34.2	39.4	42.3	47.5	50.2	53.1
15 to 15.9	403	29.6	35.0	37.9	43.3	46.2	49.0	51.7
16 to 16.9	340	30.5	33.3	36.2	41.9	44.7	50.3	53.1
17 to 17.9	240	25.7	31.5	37.4	40.3	46.0	49.1	52.0
Total	3211	32.3	35.9	39.3	42.3	46.0	49.0	52.0
**Girls**								
9 to 9.9	293	41.1	41.2	41.2	43.4	43.5	45.8	48.2
10 to 10.9	628	39.3	39.3	39.3	41.7	43.9	46.4	48.8
11 to 11.9	585	37.3	37.3	37.3	39.8	42.3	44.7	47.2
12 to 12.9	464	35.4	35.4	37.8	40.3	40.5	43.0	45.6
13 to 13.9	429	33.4	33.4	36.1	38.5	41.2	43.9	46.5
14 to 14.9	555	31.5	31.5	34.2	36.9	39.6	42.3	45.0
15 to 15.9	434	29.6	29.6	32.3	35.0	37.7	40.6	46.0
16 to 16.9	383	27.6	30.3	30.5	33.3	36.2	39.0	44.6
17 to 17.9	262	25.7	28.5	28.6	31.5	34.5	40.2	43.2
Total	4033	28.6	31.5	35.2	39.3	41.7	44.1	46.5

P = percentile.

Table S1 and Figures S1 and S2 (Supporting Information) presents altitude‐adjusted V˙O_2peak_ normative values. As we applied a systematic correction factor, the pattern of sex‐ and age‐related changes is identical as previously describe for the unadjusted data.

Finally, comparisons of the mean (±SD) for the V˙O_2peak_ (ml kg^−1^ min^−1^) from this study are presented in Table S2 and Table S3 (Supporting Information). Based on the raw non‐adjusted data, we found that Bogota boys had lower V˙O_2peak_ values than their counterparts from England, Canada, Argentina, Spain, Portugal, and Australia. Bogota schoolchildren girls had lower V˙O_2peak_ values than counterparts from England, Canada, and Australia. After performing the altitude adjustment, only youth from Canada had higher V ˙O_2peak_ values than youth in Colombia.

## 4 Discussion

This study presented for the first time smoothed reference values for the 20 m shuttle‐run performance and predicted V˙O_2peak_ (ml kg^−1^ min^−1^) among a large, population‐based sample of school‐aged youth from Bogota, Colombia. These results can be used as a baseline for long‐term physical fitness surveillance in the city, the country, and the Latin American region. The 20 m shuttle‐run performance curves can also be useful for cross‐country comparisons as efforts to include CRF testing in national health and nutrition studies and surveillance efforts gain more traction, in particular in low‐middle income countries (Barnett et al., 1995; Catley and Tomkinson, 2013; LaMonte & Blair, 2006; Matsudo & Matsudo, 1994; Ortega et al., 2005; Ortega, Ortega, Ruiz, Castillo, et al., 2008; Ortega, Ruiz, Hurtig‐Wennlof, & Sjostrom, 2008; Sandercock et al., 2012; Secchi, García, España‐Romero, & Castro‐Piñero, 2014; Slinger, Breda, & Kupiers, 2009; Tomkinson & Olds, 2007; Santos et al., 2014; Silva et al., 2012; California Physical Fitness Report; Ruiz et al., 2015).

The age‐ and gender‐related developmental patterns of aerobic fıtness have been well studied in non‐representative samples (Liu et al., 1992; Catley & Tomkinson, 2013; LaMonte & Blair, 2006; Ortega et al., 2011; Ortega, Ortega, Ruiz, Hurtig‐Wennlof, et al., 2008). Data from this study show the well‐documented differences between sexes and the typical changes in aerobic test performance associated with growth and maturation in youth (Malina, Beunen, Lefevre, & Woynarowska, 1997). For example, boys' test performance was higher than that of girls at all ages and their CRF levels remained relatively stable. In girls, it is generally thought that V ˙O_2peak_ decreases during adolescence.

We found six studies, published within the last 10‐years, using similar CRF field‐tests in population‐based samples of children and adolescents and presenting age‐ and gender‐specific normative values. Compared to their counterparts and based on our non‐adjusted V˙O_2peak_ values, Colombian boys had the lowest CRF levels found in any country with available data. Among Colombian girls, CRF levels were lower than those reported for England, Canada, and Australia but on par or higher than for Argentina, Spain, and Portugal girls. (Cole & Green, 1992; Matsudo & Matsudo, 1994; Sandercock et al., 2012; Secchi et al., 2014; Tomkinson & Olds, 2007) (Supporting Information File S4). Given that the prevalence of physical inactivity and overweight/obesity are generally lower in Colombia than in the mentioned countries (González et al. 2014; Rivera et al., 2014), such findings seemed counter‐intuitive. However, after performing the altitude adjustment, only youth from Canada had higher V˙O_2peak_ values than youth in Colombia.

At sea level, the standard barometric pressure is 760 mmHg, the oxygen partial pressure in the atmosphere is 160 mmHg and in the arteries drops to 100 mmHg. Comparatively, values at 2660 m are 559 mmHg, 100 mm Hg, and 60 mmHg for standard barometric pressure, atmospheric, and arterial blood oxygen partial pressure, respectively. A 40% lower arterial oxygen partial pressure leads to arterial oxygen desaturation (Sa0_2_ = 89%) despite adequate erythropoietic adaptations among individuals regularly living at such altitudes (Fulco et al., 1998; Squires & Buskirk, 1982). We believe that the correction factor we applied is warranted and altitude‐adjusted CRF normative values should be presented. There are no published estimates regarding the number of 10‐ to 20‐year olds living at high altitude (above 2000 m sea level). A previously published report estimated that 130 million people lived above 2000 m sea level based on global population estimates for 1994 (Cohen & Small, 1998). According to World Bank figures, the global population has grown 30% from 1994 (5.6 Billion) to 2015 (7.3 Billions) (World Bank, 2015). Assuming a steady 30% population growth for the global population living above 2000 m sea level and given that on average 9.8% of the population in low‐middle income countries are in the 10‐ to 20‐year old range, we conservatively estimated that 16,562,000 million youth between 10‐ and 20‐year old live at high altitude (World Bank, 2015). Therefore, normative CRF values such as the ones developed for this study may need to be created or adjusted for youth living at high altitudes in the Andes in Latin America and in mountain ranges in Central America, Europe, Asia, and Africa.

Even after adjusting for altitude, a significant proportion of Colombian youth (17.9% boys; 27.3% girls) showed unhealthy CRF levels according to the combined prevalence of needs improvement and health risk FITNESSGRAM categories. This proportion is similar to that reported in a representative sample of 13‐ to 17‐year olds in Chile (Garber et al., 2014) and similar population‐based samples of schoolchildren in Argentina, the UK, Sweden, and Spain, but lower than those reported in the United States, Australia, and a Pan‐European sample (Supporting Information Files S4). The prevalence of physical inactivity and sedentary behavior among children and adolescents has increased in several countries in recent decades (Ortega, Ortega, Ruiz, Hurtig‐Wennlof, et al., 2008; Rey‐López, Vicente‐Rodríguez, Biosca, & Moreno, 2008; Sandercock & Ogunleye, 2013), and consequently it is possible that physical fitness values may also have changed (Craig, Shields, Leblanc, & Tremblay, 2012). Historical declines in fitness may indeed have been partly due to concomitant increases in body mass (Olds, Ridley, & Tomkinson, 2007; Stratton et al., 2007). Sandercock, Voss, McConnell, & Rayner (2010) indicated that increases in BMI may explain some of the decrease in fitness; however, Sandercock, Ogunleye, & Voss (2015) reported an 8% decrease in fitness between 1998 and 2008 that was largely independent of changes in BMI. Support for this hypothesis was elegantly provided in the Fitlinx project, which reported declines in shuttle‐run performance concurrent with increases in BMI (Sandercock et al., 2015).

In Latin America, for example, populations have disparities in health along with disparities in modifiable risk factors, including low participation in physical activity.

Low‐middle income countries, such as Colombia, are experiencing rapid urbanization and integration with global markets. This has led to changes in diet and physical activity, which in turn have had large effects on body composition and other health‐related fitness components (González et al., 2014; Lopez, Mathers, Ezzati, Jamison, Murray, 2006; Malina et al., 1997; Parra et al., 2015; Ramírez‐Vélez et al., 2015). It is well known that CRF and muscular fitness (Buchan et al., 2015; Melo et al., 2015) are better predictors of cardiovascular disease risk factors in children than BMI, and prospective and case–control studies have shown that, even with a normal BMI, those with lower physical fitness are at increased risk of cardiovascular disease risk and premature death (Ekelund et al., 2007; Ortega, Silventoinen, Tynelius, & Rasmussen, 2012). These changes are contributing to a global increase in the prevalence of non‐communicable diseases (Malina et al., 1997). Therefore, the inclusion of CRF within health surveillance systems is justifiable and has been recommended (Kaminsky et al., 2013). Schools may be an ideal setting to monitor youth fitness (De Miguel‐Etayo et al., 2014) and could help to formulate specific strategies to promote the future health of youth (España‐Romero et al., 2010; Santos et al., 2014; Silva et al., 2012; Tovar et al., 2008; California Physical Fitness Report; Ruiz et al., 2015).

Low CRF is a strong and independent predictor of cardio‐metabolic disease in adults (Kodama et al., 2009). Mesa et al. (2006) tested CRF using the 20 m shuttle‐run and used ROC analyses to determine the cut‐ off point for aerobic fitness and an adverse blood lipid profile in adolescents (ages from 13 to 18 years). Lobelo, Pate, Dowda, Liese, & Daniels (2009) reported that the CRF thresholds that best discriminated between low and high cardio‐metabolic risk in a representative sample of U.S. adolescents were very similar to those established in 2004 by the FITNESSGRAM^®^ expert panel. A similar method was then used by the FITNESSGRAM group to develop their current health‐related standards (Welk et al., 2011). These data can be useful to guide existing noncommunicable disease prevention efforts in Colombia (Aguilar et al., 2011; González et al., 2014). The fact that almost 1 in 5 Colombian boys and 1 in 4 girls had a low aerobic capacity, suggestive of an increased future cardio‐metabolic risk is noteworthy. However, these values should be interpreted with caution, as the data have been converted to predicted V˙O_2peak_ based on one of a number of available reference equations, which has limitations (Aguilar et al., 2011; Sandercock et al., 2012). We adopted the 20 m shuttle‐run test to estimate CRF because it has been extensively used in situations when it is unfeasible to use direct measurements (Machado‐Rodrigues et al., 2014; Moreira et al., 2011; Prieto‐Benavides et al., 2015), and because its results are highly correlated with laboratory measurements (Batista et al., 2013).

There are several limitations to this study, leading us to be cautious lest we over‐interpret these findings. First, we did not measure important variables in relation to CRF and altitude, such as levels of physical activity, sex hormone levels, family background birthplace, and time living in Bogota. Second, the estimation of V˙O_2peak_ from the 20 m shuttle‐run is known to vary according to the equation used. A previous study (Boiarskaia, Boscolo, Zhu, & Mahar, 2011) has tested the degree of agreement between various equations used to estimate V˙O_2peak_ and the actual V˙O_2peak_. The equation used to estimate V˙O_2peak_ in this study may have underestimated CRF by up to 12% relative to other methods and therefore may have, in isolation, inflated the prevalence of unhealthy aerobic capacity (Leger et al., 1988; Sandercock et al., 2012). Therefore, we considered our low CRF estimates to be conservative. Third, this study includes participants from public schools in one city, and thus the data are not fully representative of the full population in the city or the country. However, Bogota is the largest urban center in Colombia comprising about 15% of the country's population. It includes a mix of locally born residents and populations from other regions of the country that relocate there with large racial and cultural diversity. Another limitation is that this study did not include assessments of students attending private schools. This because the study was deployed in collaboration with the Bogota District Education Department, which only has jurisdiction among public schools. However, the public system constitutes the majority of school offering in the city, with 85% of school‐age children enrolled in the city public school system. Therefore, inferences to all Bogota or Colombian children and adolescents should be made cautiously. Future population‐based studies collecting data for nationally representative samples, such as the one recently conducted in Chile, are still needed in Colombia and other countries in the region (Lobelo et al., 2009; Moreira et al., 2011; Ruiz et al., 2015; Sandercock et al., 2012).

Conversely, our decision to categorize CRF according to its health predictive value instead of using continuous variables can be considered a strength of the study, as it allowed for greater public health interpretability. Taking advantage of a newly compiled and large population‐based sample, this study develops centile references for Colombian schoolchildren using the popular LMS method (Cole & Green, 1992) and smoothed and specific CRF curves for each age and gender are further generated from LMS model parameters to facilitate direct comparison of Colombian data with international references. The results contribute to the recent application of the LMS statistical procedure for the construction of growth percentiles for a variety of outcomes (e.g., BMI, waist circumference, blood pressure) (Carrel et al., 2012; Eisenmann & Malina, 2002; Haugen et al., 2014; Pate et al., 2006; Roriz De Oliveira et al., 2014; Sandercock et al., 2012; Tomkinson et al., 2007). Another potential strength of the study was the use of valid and reliable field tests recommended for Latin American youth school‐based fitness assessment.

In conclusion, our results provide sex‐ and age‐specific normative reference standards for the 20 m shuttle‐run test and estimated V˙O_2peak_ values in a large, population‐based sample of schoolchildren from Bogota, a city situated at high altitude. These curves can be used as a reference with which to compare the performance of individuals of a corresponding age in the city, country, and region. Establishing these reference percentiles allows for comparison of CRF in schoolchildren, who vary geographically and demographically, with others in similar settings. The reference percentiles also enable year‐by‐year tracking of the fitness profiles of large numbers of students now participating in statewide school‐based initiatives to improve fitness. We also introduce a method to adjust V˙O_2peak_ levels to account for the impact of high altitude on CRF assessment that can be replicated in future studies. Future research aimed at establishing the CRF cut‐off value associated with high cardio‐metabolic risk in this population is warranted.

## Supporting information

Supporting Information Figure 1.

Supporting Information Figure 2.

Supporting Information Table 1.

Supporting Information Table 2.

Supporting Information Table 3.
