# Supplementary figures and images for: Normative reference values for the 20 m shuttle‐run test in a population‐based sample of school‐aged youth in Bogota, Colombia: the FUPRECOL study
Source: Am J Hum Biol. 2016 Aug 8;29(1):e22902. doi: 10.1002/ajhb.22902 (PMC5298048; doi:10.1002/ajhb.22902)

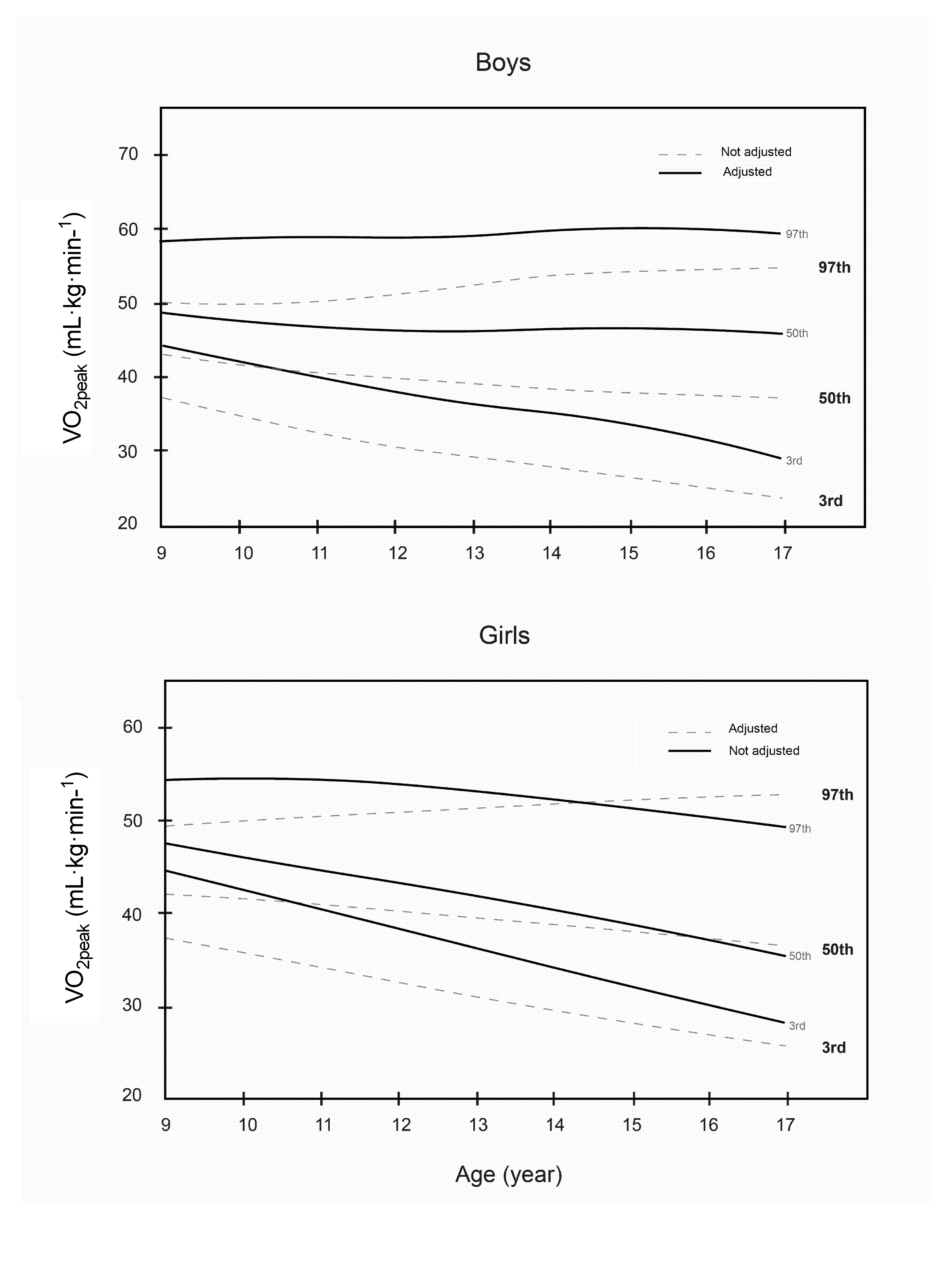

Supplement: Supplementary file 2 — Supporting Information Figure 2. [file AJHB-29-0-s002.tif]
