# Supplementary material for: Normative reference values for the 20 m shuttle‐run test in a population‐based sample of school‐aged youth in Bogota, Colombia: the FUPRECOL study
Source: Am J Hum Biol. 2016 Aug 8;29(1):e22902. doi: 10.1002/ajhb.22902 (PMC5298048; doi:10.1002/ajhb.22902)
Supplement: Supplementary file 3 — Supporting Information Table 1. [file AJHB-29-0-s003.docx]

**Supplemental File Table S1.** Smoothed age- and sex-specific percentile values for VO_2peak_ (ml•kg^-1^•min^-1^) (altitude-adjusted) among a population-based sample of schoolchildren in Bogota, Colombia

|  | n | M | SD | P_3_ | P_10_ | P_25_ | P_50_ | P_75_ | P_90_ | P_97_ |
| --- | --- | --- | --- | --- | --- | --- | --- | --- | --- | --- |
| **Boys** |  |  |  |  |  |  |  |  |  |  |
| 9 to 9.9 | 215 | 49.5 | 3.5 | 45.6 | 45.7 | 45.7 | 48.3 | 50.8 | 53.5 | 57.3 |
| 10 to 10.9 | 399 | 48.4 | 4.1 | 43.6 | 43.6 | 46.1 | 48.7 | 51.5 | 54.2 | 56.8 |
| 11 to 11.9 | 408 | 47.1 | 4.4 | 41.4 | 41.4 | 44.2 | 47.0 | 49.6 | 52.6 | 55.2 |
| 12 to 12.9 | 381 | 46.4 | 5.1 | 39.3 | 39.3 | 42.1 | 45.0 | 50.4 | 53.4 | 56.2 |
| 13 to 13.9 | 391 | 46.6 | 5.6 | 37.1 | 39.9 | 43.0 | 45.8 | 48.7 | 54.6 | 57.5 |
| 14 to 14.9 | 434 | 47.0 | 6.5 | 35.0 | 38.0 | 43.8 | 47.0 | 52.7 | 55.7 | 58.9 |
| 15 to 15.9 | 403 | 46.8 | 6.8 | 32.9 | 38.8 | 42.1 | 48.0 | 51.3 | 54.4 | 57.4 |
| 16 to 16.9 | 340 | 46.5 | 6.9 | 33.9 | 37.0 | 40.2 | 46.5 | 49.6 | 55.8 | 58.9 |
| 17 to 17.9 | 240 | 45.4 | 7.1 | 28.5 | 35.0 | 41.5 | 44.7 | 51.1 | 54.5 | 57.7 |
| *Total* | *3211* | *47.1* | *5.8* | *35.9* | *39.9* | *43.6* | *47.0* | *51.1* | *54.4* | *57.7* |
| **Girls** |  |  |  |  |  |  |  |  |  |  |
| 9 to 9.9 | 293 | 48.0 | 2.6 | 45.6 | 45.7 | 45.7 | 48.2 | 48.3 | 50.8 | 53.5 |
| 10 to 10.9 | 628 | 46.6 | 3.2 | 43.6 | 43.6 | 43.6 | 46.3 | 48.7 | 51.5 | 54.2 |
| 11 to 11.9 | 585 | 45.0 | 3.2 | 41.4 | 41.4 | 41.4 | 44.2 | 47.0 | 49.6 | 52.4 |
| 12 to 12.9 | 464 | 43.7 | 3.4 | 39.3 | 39.3 | 41.9 | 44.8 | 45.0 | 47.7 | 50.6 |
| 13 to 13.9 | 429 | 42.8 | 4.4 | 37.1 | 37.1 | 40.1 | 42.8 | 45.7 | 48.7 | 51.6 |
| 14 to 14.9 | 555 | 41.3 | 4.4 | 35.0 | 35.0 | 38.0 | 41.0 | 44.0 | 47.0 | 50.0 |
| 15 to 15.9 | 434 | 39.1 | 4.7 | 32.9 | 32.9 | 35.9 | 38.8 | 41.9 | 45.1 | 51.1 |
| 16 to 16.9 | 383 | 37.9 | 5.0 | 30.6 | 33.7 | 33.9 | 37.0 | 40.2 | 43.3 | 49.5 |
| 17 to 17.9 | 262 | 36.3 | 5.3 | 28.5 | 31.6 | 31.7 | 35.0 | 38.3 | 44.6 | 48.0 |
| *Total* | *4033* | 42.7 | 5.3 | 31.7 | 35.0 | 39.1 | 43.6 | 46.3 | 49.0 | 51.6 |

M, mean; SD, standard deviation; P, percentile
