# Supplementary material for: Normative reference values for the 20 m shuttle‐run test in a population‐based sample of school‐aged youth in Bogota, Colombia: the FUPRECOL study
Source: Am J Hum Biol. 2016 Aug 8;29(1):e22902. doi: 10.1002/ajhb.22902 (PMC5298048; doi:10.1002/ajhb.22902)
Supplement: Supplementary file 4 — Supporting Information Table 2. [file AJHB-29-0-s004.docx]

**Supplemental File Table S2.** Reference values for VO_2peak_ (ml•kg^-1^•min^-1^) mean (± SD) from selected studies

| Study | FUPRECOL Study (2015)  n = 7244 | Altitude-adjusted FUPRECOL  Study (2015)  n = 7244 | England  (Sandercock et al., 2012)  n = 7366 | Canada  (Tremblay et al., 2010)  n = 7000 | Argentina  (Secchi et al., 2014)  n = 1867 | Spain  (Ortega et al., 2005)  n = 3528 | Portugal  (Santos et al., 2014)  n = 22048 | Australia  (Tomkinson et al 2007)  n = 85347 |
| --- | --- | --- | --- | --- | --- | --- | --- | --- |
| **Boys** |  |  |  |  |  |  |  |  |
| 9 to 9.9 | 44.6 (3.2) | 49.5 (3.5) | - | 51.5 (4.3) | - | - | - | 43.4 (4.6) |
| 10 to 10.9 | 43.6 (3.7) | 48.4 (4.1) | 47.1 (5.6) | 51.6 (4.2) | - | - | 43.9 (2.4) | 43.9 (4.8) |
| 11 to 11.9 | 42.4 (4.0) | 47.1 (4.4) | 45.9 (5.4) | 51.1 (4.5) | 44.6 (5.2) | - | 44.6 (4.9) | 44.6 (4.9) |
| 12 to 12.9 | 41.8 (4.6) | 46.4 (5.1) | 45.3 (5.8) | 51.9 (5.1) | 45.4 (5.1) | - | 42.9 (5.1) | 42.9 (5.1) |
| 13 to 13.9 | 42.0 (5.0) | 46.6 (5.6) | 45.2 (6.2) | 50.0 (5.2) | 45.4 (5.1) | 43.8 (5.4) | 43.8 (5.3) | 41.1 (5.3) |
| 14 to 14.9 | 42.4 (5.9) | 47.0 (6.5) | 46.3 (6.9) | 50.1 (5.2) | 42.1 (5.4) | 44.8 (5.4) | 44.8 (5.4) | 42.1 (5.4) |
| 15 to 15.9 | 42.2 (6.2) | 46.8 (6.8) | 45.7 (7.1) | 50.2 (6.0) | 43.3 (5.5) | 43.3 (5.5) | 43.3 (5.5) | 43.3 (5.5) |
| 16 to 16.9 | 41.9 (6.3) | 46.5 (6.9) | 46.4 (5.7) | 49.9 (5.8) | 44.6 (5.7) | 41.7 (5.4) | 44.6 (5.7) | 44.6 (6.7) |
| 17 to 17.9 | 40.9 (6.4) | 45.4 (7.1) | - | 49.6 (5.9) | 40.2 (5.9) | 43.1 (5.8) | 43.1 (5.8) | 44.5 (5.8) |
| *Total* | *42.4* (*5.2*) | *47.1* (*5.8*) | *45.8 (6.2)* |  | *43.7 (5.4)* | *43.3 (5.5)* | *43.9 (5.0)* | *43.4 (5.3)* |
| **Girls** |  |  |  |  |  |  |  |  |
| 9 to 9.9 | 43.3 (2.4) | 48.0 (2.6) | - | 49.9 (3.2) | - |  | - | 41.1 (4.6) |
| 10 to 10.9 | 42.0 (2.9) | 46.6 (3.2) | 44.7 (4.6) | 46.8 (2.7) | - |  | 41.5 (4.8) | 41.5 (4.8) |
| 11 to 11.9 | 40.5 (2.9) | 45.0 (3.2) | 43.0 (4.3) | 47.5 (4.0) | 42.1 (5.1) |  | 42.1 (5.0) | 39.6 (4.9) |
| 12 to 12.9 | 39.4 (3.1) | 43.7 (3.4) | 41.9 (4.4) | 46.6 (4.1) | 40.3 (5.1) |  | 40.3 (5.1) | 40.3 (5.1) |
| 13 to 13.9 | 38.5 (4.0) | 42.8 (4.4) | 40.7 (4.7) | 44.4 (4.7) | 38.5 (5.2) | 38.5 (5.2) | 38.5 (6.0) | 38.5 (5.2) |
| 14 to 14.9 | 37.2 (4.0) | 41.3 (4.4) | 39.2 (5.0) | 41.6 (4.7) | 36.7 (5.4) | 36.7 (5.4) | 36.7 (5.4) | 37.8 (5.2) |
| 15 to 15.9 | 35.2 (4.2) | 39.1 (4.7) | 37.2 (4.7) | 41.1 (5.0) | 35.0 (5.5) | 35.0 (5.5) | 35.0 (5.2) | 37.5 (4.8) |
| 16 to 16.9 | 34.1 (4.5) | 37.9 (5.0) | 37.8 (5.8) | 39.5 (4.9) | 33.2 (5.7) | 33.2 (6.3) | 36.0 (5.7) | 38.2 (5.1) |
| 17 to 17.9 | 32.7 (4.8) | 36.3 (5.3) | - | 38.6 (5.1) | 32.4 (5.8) | 32.4 (5.8) | 34.3 (5.8) | 34.3 (5.8) |
| *Total* | *38.5* (*4.8*) | *42.7 (5.3)* | *41.2 (5.0)* |  | *36.5 (5.4)* | *35.2 (5.6)* | *38.5 (5.4)* | *38.8 (5.1)* |
