# Supplementary material for: Normative reference values for the 20 m shuttle‐run test in a population‐based sample of school‐aged youth in Bogota, Colombia: the FUPRECOL study
Source: Am J Hum Biol. 2016 Aug 8;29(1):e22902. doi: 10.1002/ajhb.22902 (PMC5298048; doi:10.1002/ajhb.22902)
Supplement: Supplementary file 5 — Supporting Information Table 3. [file AJHB-29-0-s005.docx]

**Supplemental File Table S3.** Prevalence of unhealthy CRF among Colombian schoolchildren and selected comparable population-based studies in other countries**.**

| **Location** | **Sample year** | **n** | **Age sample** | **Health-related CRF Standard** | **Percent unhealthy^c^** | |
| --- | --- | --- | --- | --- | --- | --- |
|  |  |  |  |  | **Boys** | **Girls** |
| South America |  |  |  |  |  |  |
| Fuprecol Study^a^ | 2015 | 7,244 | 9 to 17 | FITNESSGRAM 2011 | 45% | 59% |
| Altitude-adjusted Fuprecol Study^b^ | 2015 | 7,244 | 9 to 17 | FITNESSGRAM 2011 | 17% | 27% |
| Argentina^1^ | 2014 | 1,867 | 6 to 19 | FITNESSGRAM 2011 | 11% | 49% |
| Chile (R)^2^ | 2014 | 19,904 | 13 to 17 | FITNESSGRAM 2011 | 15% | 30% |
| Colombia (Bogotá)^3^ | 2008 | 665 | 7 to 18 | FITNESSGRAM 2004 | 37% | - |
| Colombia (Cali)^4^ | 2011 | 1,773 | 10 to 16 | FITNESSGRAM 2004 | 52% | 60% |
| USA |  |  |  |  |  |  |
| California^5^ | 2013 | 1,666 | 11 to 18 | FITNESSGRAM 2011 | 28% | 44% |
| Midwest^6^ | 2011 | 447,619 | 8 to 17 | FITNESSGRAM 2011 | 26% | 23% |
| USA – NHANES (R)^7^ | 1999-2002 | 1,247 | 12 to 19 | FITNESSGRAM 2004 | 35% | 35% |
| Europe |  |  |  |  |  |  |
| UK (East)^8^ | 2013 | 7,366 | 10 to 16 | FITNESSGRAM 2011 | 12% | 25% |
| Sweden (R)^9^ | 2008 | 472 | 14 to 16 | FITNESSGRAM 2004 | 9% | 20% |
| Spain (R)^10^ | 2005 | 1,867 | 14 to 16 | FITNESSGRAM 2004 | 19% | 17% |
| Pan-European (R)^11^ | 2008 | 3,428 | 12 to 17 | FITNESSGRAM 2004 | 39% | 43% |
| Australia^12^ | 1985-2009 | 18,075 | 9 to 17 | FITNESSGRAM 2004 | 29% | 23% |

^a^ V˙O_2peak_ (ml•kg^-1^•min^-1^) predicted using the equation of Leger et al. (1988)

^b^ V˙O_2peak_ (ml•kg^-1^•min^-1^) predicted using the equation of Leger et al. (1988) and adjusted by correction factor (1.11) and 2011 FITNESSGRAM^®^ standards and Healthy Fitness Zones (Welk et al., 2011)

^a^ V˙O_2peak_ (ml•kg^-1^•min^-1^) predicted using the Leger et al. equation (1988) and adjusted by correction factor (1.11)

^b^ To classify VO_2peak_, we used the 2011 FITNESSGRAM^®^ standards and Healthy Fitness Zones (Welk et al., 2011)

^c^ If three categories reported, unhealthy combines the two higher-risk categories. (R) Sample is nationally representative.

Reference

1. Secchi D, García C, España-Romero V, Castro-Piñero J. (2014). Condición Física y riesgo cardiovascular futuro en niños y adolescentes argentinos:una introducción de la bateria ALPHA. Arch Argent Pediatr, 112(2), 132-140.
2. Garber MD, Sajuria M, Lobelo F. (2014). Geographical variation in health-related physical fitness and body composition among Chilean 8th graders: a nationally representative cross-sectional study. Plos One, 9, e108053.
3. Tovar G, Poveda JG, Pinilla MI, Lobelo F. (2008). Sobrepeso, inactividad física y baja condición física en un colegio de Bogotá, Colombia [Relationship between overweight, physical activity and physical fitness in school-aged boys in Bogota Colombia]. Arch Latinoam Nutr, 58, 265-273.
4. Aguilar AC, Pradilla A, Mosquera M, Gracia AB, Ortega JG, Leiva JH, et al. (2011). Percentile values for physical condition for Cali, Colombian children and adolescents. Biomedica, 31, 242-248.
5. California Physical Fitness Report. Sacramento, CA: California Department of Education. Available: http://data1.cde.ca.gov/dataquest/PhysFitness/PFTDN/Summary2011.aspx?r=0&t=4&y=2012-13&c=00000000000000&n=0000 Accessed 1 Dec 2015.
6. Welk GJ, De Saint-Maurice Maduro PF, Laurson KR, Brown DD. (2011). Field Evaluation of the New FITNESSGRAM Criterion-Referenced Standards. Am J Prev Med, 41, S131-142.
7. Pate RR, Wang CY, Dowda M, Farrell SW, O’Neill JR. (2006). Cardiorespiratory fitness levels among US youth 12 to 19 years of age: findings from the 1999–2002 National Health and Nutrition Examination Survey. Arch Pediatr Adolesc Med, 160, 1005-1012.
8. Sandercock G, Voss C, Cohen D, Taylor M, Stasinopoulos DM. (2012). Centile curves and normative values for the twenty metre shuttle-run test in English schoolchildren. J Sports Sci, 30(7), 679-87.
9. Ortega FB, Ruiz JR, Hurtig-Wennlof A, Sjostrom M. (2008b). Physically active adolescents are more likely to have a healthier cardiovascular fitness level independently of their adiposity status. The European youth heart study. Rev Esp Cardiol, 61, 123-129.
10. Ortega FB, Ruiz JR, Castillo MJ, Moreno LA, González-Gross M, Wärnberg J, et al. (2005). Low level of physical fitness in Spanish adolescents. Relevance for future cardiovascular health: AVENA study. Rev Esp Cardiol, 58(8): 898-909.
11. Ortega FB, Artero EG, Ruiz JR, España-Romero V, Jiménez-Pavón D, Vicente-Rodriguez G., et al., (2011). Physical fitness levels among European adolescents: the HELENA study. Br J Sports Med, 45, 20-29.
12. Catley MJ, Tomkinson GR. (2013). Normative health-related fitness values for children: analysis of 85347 test results on 9-17-year-old Australians since 1985. Br J Sports Med, 47, 98-108.
